# Supplementary material for: Body Composition Assessment in Mexican Children and Adolescents. Part 2: Cross-Validation of Three Bio-Electrical Impedance Methods against Dual X-ray Absorptiometry for Total-Body and Regional Body Composition
Source: Nutrients. 2022 Feb 25;14(5):965. doi: 10.3390/nu14050965 (PMC8912617; doi:10.3390/nu14050965)
Supplement: Supplementary file 1 [file nutrients-14-00965-s001.zip › nutrients-1604578-supplementary.pdf]

Supplementary Figure S1. Position of the electrodes for BIA supine position.

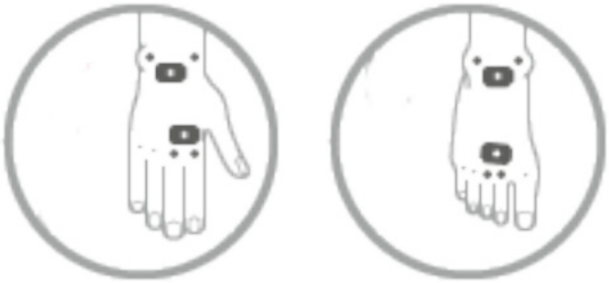

Supplementary Tables.

Supplementary Table S1: BIA standing measurements. Relative importance of the impedance "data" in predicting body composition.

predictor	R^2	SEE		$R^2_{absolute}$	$SEE_{absolute}$
**TBW / weight, L/kg**				**TBW, L**	
height^2 / (R_{50} · weight), cm^2 / (Ω kg)	0.510	0.0570	$p < 0.001$	0.913	2.47
Xc_{50}, Ω	0.569	0.0535	$p < 0.001$	0.927	2.28
Index $R_{50\ trunk/extremities}$	0.580	0.0530	$p < 0.05$	0.930	2.24
R_{50} / weight, Ω / kg	0.585	0.0527	$p = 0.10$	0.930	2.24
intercept			$p = 0.25$		
**FFM / weight, %**				**FFM, kg**	
height^2 / (R_{50} · weight), cm^2 / (Ω kg)	0.654	5.71	$p < 0.001$	0.953	2.52
Xc_{50}, Ω	0.729	5.06	$p < 0.001$	0.966	2.15
Index $R_{50\ trunk/extremities}$	0.752	4.86	$p = 0.07$	0.969	2.04
R_{50} / weight, Ω / kg	0.756	4.83	$p < 0.001$	0.968	2.08
weight, kg	0.770	4.70	$p < 0.001$	0.971	2.02
Index $Xc_{50\ trunk/extremities}$	0.772	4.68	$p = 0.10$	0.971	2.02
intercept			$p = 0.43$		
**$FM_{trunk\ DXA}$ / weight, %**				**$FM_{trunk\ DXA}$, kg**	
height^2 / ($R_{50\ trunk}$ · weight), cm^2 / (Ω kg)	0.779	2.53	$p < 0.001$	0.934	1.209
Index $R_{50\ trunk/extremities}$	0.864	1.98	$p < 0.001$	0.957	0.985
waist circumference^2, cm^2	0.875	1.90	$p = 0.15$	0.961	0.931
weight, kg	0.897	1.73	$p < 0.001$	0.967	0.856
gender	0.907	1.64	$p < 0.001$	0.970	0.824
age, month	0.910	1.62	$p < 0.01$	0.971	0.805
waist circumference, cm	0.916	1.56	$p < 0.001$	0.974	0.766
$Xc_{50\ trunk}$, Ω	0.918	1.55	$p < 0.001$	0.975	0.754
Index $Xc_{50\ trunk/extremities}$	0.926	1.46	$p < 0.001$	0.977	0.724
intercept			$p < 0.001$		
**$LST_{right\ arm}$ / weight, %**				**$LST_{right\ arm}$, kg**	
height^2 / ($R_{50\ right\ arm}$ · weight), cm^2 / (Ω kg)	0.458	0.446	$p < 0.001$	0.897	0.236
height, cm	0.786	0.280	$p < 0.001$	0.960	0.147
gender	0.805	0.268	$p < 0.001$	0.964	0.139
age, month	0.817	0.259	$p < 0.001$	0.967	0.133
weight, kg	0.824	0.254	$p < 0.001$	0.969	0.130
$Xc_{50\ right\ arm}$, Ω	0.845	0.239	$p < 0.001$	0.972	0.123
$R_{50\ right\ arm}$, Ω	0.851	0.234	$p < 0.001$	0.975	0.116
Index $R_{50\ trunk/extremities}$	0.863	0.224	$p < 0.001$	0.977	0.112
Index $Xc_{50\ trunk/extremities}$	0.864	0.223	$p < 0.001$	0.977	0.112
intercept			$p = 0.61$		
**$LST_{left\ arm}$ / weight, %**				**$LST_{left\ arm}$, kg**	
height^2 / ($R_{50\ left\ arm}$ · weight), cm^2 / (Ω kg)	0.444	0.426	$p < 0.001$	0.890	0.231
weight, kg	0.772	0.273	$p < 0.001$	0.956	0.146
$Xc_{50\ left\ arm}$, Ω	0.812	0.248	$p < 0.001$	0.965	0.131
gender	0.824	0.240	$p < 0.001$	0.966	0.128
Index $R_{50\ trunk/extremities}$	0.833	0.234	$p < 0.001$	0.968	0.125
$R_{50\ left\ arm}$, Ω	0.842	0.228	$p < 0.001$	0.971	0.119
height, cm	0.848	0.223	$p < 0.001$	0.973	0.114

age, month	0.849	0.222	$p < 0.001$	0.974	0.113
Index $Xc_{50\ trunk/extremities}$	0.850	0.222	$p < 0.001$	0.974	0.112
intercept			$p = 0.72$		
**$LST_{right\ leg}$ / weight, %**				**$LST_{right\ leg}$, kg**	
height² / ($R_{50\ right\ leg}$ · weight), cm² / (Ω kg)	0.278	1.288	$p < 0.001$	0.923	0.629
height, cm	0.773	0.723	$p < 0.001$	0.974	0.368
$Xc_{50\ right\ leg}$, Ω	0.822	0.641	$p < 0.001$	0.980	0.321
$R_{50\ right\ leg}$, Ω	0.836	0.614	$p < 0.001$	0.982	0.302
Index $R_{50\ trunk/extremities}$	0.849	0.591	$p < 0.001$	0.984	0.290
gender	0.852	0.584	$p < 0.001$	0.984	0.287
weight, kg	0.853	0.582	$p < 0.001$	0.984	0.285
age, month	0.854	0.580	$p < 0.001$	0.984	0.284
Index $Xc_{50\ trunk/extremities}$	0.855	0.579	$p < 0.01$	0.984	0.283
intercept			$p = 0.57$		
**$LST_{left\ leg}$ / weight, %**				**$LST_{left\ leg}$, kg**	
height² / ($R_{50\ left\ leg}$ · weight), cm² / (Ω kg)	0.269	1.285	$p < 0.001$	0.922	0.625
height, cm	0.777	0.710	$p < 0.001$	0.974	0.360
$Xc_{50\ left\ leg}$, Ω	0.822	0.635	$p < 0.001$	0.980	0.316
$R_{50\ left\ leg}$, Ω	0.837	0.608	$p < 0.001$	0.982	0.298
Index $R_{50\ trunk/extremities}$	0.848	0.586	$p < 0.001$	0.984	0.287
gender	0.851	0.580	$p < 0.001$	0.984	0.284
weight, kg	0.853	0.577	$p < 0.001$	0.984	0.282
age, month	0.854	0.576	$p < 0.001$	0.984	0.280
Index $Xc_{50\ trunk/extremities}$	0.855	0.574	$p < 0.001$	0.985	0.278
intercept			$p = 0.59$		

Values without segment indication are mean values of the right and left side of the body. SEE, standard error of estimate. R^2 and SEE are given for each step of a stepwise regression, while *p* is given for each coefficient in the final formula. R^2absolute and SEEabsolute compare results of each step of the regression with the absolute value of the reference measurement (not relative to body weight).

Supplementary Table S2: BIA supine measurements with 8 electrodes

predictor	R^2	SEE		$R^2_{absolute}$	$SEE_{absolute}$
**TBW / weight, l/kg**				**TBW, l**	
height² / (R_{50} · weight), cm² / (Ω kg)	0.480	0.0581	$p < 0.001$	0.913	2.50
Xc_{50}, Ω	0.526	0.0556	$p < 0.01$	0.925	2.32
Index $R_{50\ trunk/extremities}$	0.545	0.0546	$p < 0.01$	0.929	2.26
R_{50} / weight, Ω / kg	0.554	0.0542	$p < 0.05$	0.930	2.25
gender	0.561	0.0539	$p = 0.07$	0.931	2.24
intercept			$p < 0.01$		
**FFM / weight, %**				**FFM, kg**	
height² / (R_{50} · weight), cm² / (Ω kg)	0.651	5.69	$p < 0.001$	0.953	2.52
Xc_{50}, Ω	0.726	5.05	$p < 0.001$	0.967	2.13
Index $R_{50\ trunk/extremities}$	0.744	4.89	$p < 0.001$	0.970	2.03
R_{50} / weight, Ω / kg	0.752	4.83	$p < 0.001$	0.969	2.06
weight, kg	0.759	4.77	$p < 0.01$	0.971	2.02
intercept			$p = 0.77$		
**$FM_{trunk\ DXA}$ / weight, %**				**$FM_{trunk\ DXA}$, kg**	
height² / ($R_{50\ trunk}$ · weight), cm² / (Ω kg)	0.818	2.32	$p < 0.001$	0.949	1.073
Index $R_{50\ trunk/extremities}$	0.889	1.81	$p < 0.001$	0.966	0.878
$Xc_{50\ trunk}$, Ω	0.904	1.69	$p < 0.001$	0.971	0.815
Index $Xc_{50\ trunk/extremities}$	0.910	1.64	$p < 0.001$	0.972	0.795
waist circumference, cm	0.913	1.61	$p < 0.001$	0.972	0.789
weight, kg	0.925	1.50	$p < 0.001$	0.977	0.718
gender	0.933	1.42	$p < 0.001$	0.979	0.694
$R_{50\ trunk}$ / weight, Ω / kg	0.934	1.41	$p < 0.001$	0.979	0.694
intercept			$p < 0.001$		
**$LST_{right\ arm}$ / weight, %**				**$LST_{right\ arm}$, kg**	
height² / ($R_{50\ right\ arm}$ · weight), cm² / (Ω kg)	0.520	0.416	$p < 0.001$	0.909	0.219
height, cm	0.799	0.269	$p < 0.001$	0.961	0.142
gender	0.816	0.258	$p < 0.001$	0.965	0.135
age, month	0.826	0.251	$p < 0.001$	0.968	0.130
$Xc_{50\ right\ arm}$, Ω	0.831	0.247	$p < 0.001$	0.968	0.129

$R_{50\ right\ arm}$, Ω	0.851	0.232	$p < 0.001$	0.973	0.119
Index $R_{50\ trunk/extremities}$	0.861	0.225	$p < 0.001$	0.975	0.115
Index $Xc_{50\ trunk/extremities}$	0.862	0.224	$p < 0.001$	0.975	0.114
weight, kg	0.863	0.223	$p < 0.001$	0.975	0.114
intercept			$p < 0.05$		
**$LST_{left\ arm}$ / weight, %**				**$LST_{left\ arm}$, kg**	
height² / ($R_{50\ left\ arm}$ · weight), cm² / (Ω kg)	0.541	0.384	$p < 0.001$	0.909	0.207
weight, kg	0.796	0.256	$p < 0.001$	0.961	0.136
$Xc_{50\ left\ arm}$, Ω	0.829	0.235	$p < 0.001$	0.967	0.124
gender	0.844	0.225	$p < 0.001$	0.969	0.120
Index $R_{50\ trunk/extremities}$	0.852	0.219	$p < 0.001$	0.971	0.117
$R_{50\ left\ arm}$, Ω	0.861	0.212	$p < 0.001$	0.974	0.111
height, cm	0.864	0.210	$p < 0.001$	0.975	0.109
Index $Xc_{50\ trunk/extremities}$	0.865	0.209	$p < 0.001$	0.975	0.108
age, month	0.865	0.209	$p < 0.01$	0.975	0.108
intercept			$p < 0.01$		
**$LST_{right\ leg}$ / weight, %**				**$LST_{right\ leg}$, kg**	
height² / ($R_{50\ right\ leg}$ · weight), cm² / (Ω kg)	0.352	1.216	$p < 0.001$	0.929	0.593
height, cm	0.772	0.722	$p < 0.001$	0.973	0.366
$Xc_{50\ right\ leg}$, Ω	0.809	0.661	$p < 0.001$	0.978	0.330
gender	0.820	0.641	$p < 0.001$	0.980	0.317
Index $R_{50\ trunk/extremities}$	0.828	0.627	$p < 0.001$	0.981	0.310
$R_{50\ right\ leg}$, Ω	0.839	0.607	$p < 0.001$	0.982	0.297
Index $Xc_{50\ trunk/extremities}$	0.841	0.603	$p < 0.001$	0.983	0.294
weight, kg	0.841	0.603	$p = 0.38$	0.983	0.295
intercept			$p = 0.65$		
**$LST_{left\ leg}$ / weight, %**				**$LST_{left\ leg}$, kg**	
height² / ($R_{50\ left\ leg}$ · weight), cm² / (Ω kg)	0.352	1.201	$p < 0.001$	0.930	0.582
height, cm	0.778	0.703	$p < 0.001$	0.975	0.352
$Xc_{50\ left\ leg}$, Ω	0.810	0.651	$p < 0.001$	0.979	0.322
gender	0.823	0.628	$p < 0.001$	0.981	0.307
Index $R_{50\ trunk/extremities}$	0.831	0.615	$p < 0.001$	0.981	0.302
$R_{50\ left\ leg}$, Ω	0.841	0.596	$p < 0.001$	0.983	0.291
Index $Xc_{50\ trunk/extremities}$	0.845	0.589	$p < 0.001$	0.983	0.287
weight, kg	0.845	0.589	$p = 0.15$	0.983	0.287
intercept			$p = 0.37$		

Supplementary Table S3: mBIA – supine measurements with 4 electrodes

predictor	R^2	SEE		$R^2_{absolute}$	$SEE_{absolute}$
**TBW / weight, l/kg**				**TBW, l**	
height² / ($R_{50\ right\ side}$ · weight), cm² / (Ω kg)	0.482	0.0578	$p < 0.001$	0.917	2.45
BMI, kg/m²	0.535	0.0549	$p < 0.001$	0.928	2.29
$Xc_{50\ right\ side}$, Ω	0.550	0.0541	$p < 0.01$	0.932	2.24
intercept			$p < 0.01$		
**FFM / weight, %**				**FFM, kg**	
height² / ($R_{50\ right\ side}$ · weight), cm² / (Ω kg)	0.651	5.67	$p < 0.001$	0.956	2.47
$Xc_{50\ right\ side}$, Ω	0.719	5.09	$p < 0.001$	0.968	2.10
$R_{50\ right\ side}$ / weight, Ω / kg	0.735	4.95	$p < 0.001$	0.968	2.12
height, cm	0.743	4.89	$p < 0.01$	0.969	2.08
intercept			$p < 0.05$		
**$FM_{trunk\ DXA}$ / weight, %**				**$FM_{trunk\ DXA}$, kg**	
height² / ($R_{50\ right\ side}$ · weight), cm² / (Ω kg)	0.799	2.43	$p < 0.001$	0.936	1.200
$Xc_{50\ right\ side}$, Ω	0.860	2.02	$p < 0.001$	0.958	0.973
$R_{50\ right\ side}$, Ω	0.872	1.94	$p < 0.001$	0.960	0.947
weight, kg	0.884	1.85	$p < 0.001$	0.963	0.911
waist circumference², cm²	0.892	1.78	$p = 0.15$	0.967	0.863
gender	0.900	1.72	$p < 0.001$	0.968	0.851
waist circumference, cm	0.900	1.71	$p < 0.001$	0.968	0.847
age, month	0.901	1.71	$p < 0.05$	0.968	0.845

intercept	$p < 0.001$

Supplementary Table S4: Characteristics of the subjects measured by each BIA device in phase two.

	mBIA Handle	mBIA Handrail	mBIA supine 8e	mBIA supine 4e	*p*
Total (*n*, %)	425	297	444	449	
female	210, 49%	139, 47%	220, 50%	225, 50%	
male	215, 51%	158, 53%	224, 50%	224, 50%	0.4226
Age (years)	12.0 ± 3.6	13.7 ± 2.8*	12.1 ± 3.6	12.0 ± 3.7	< 0.0001
Weight (kg)	50.7 ± 20.3	59.5 ± 16.7*	51.2 ± 20.2	50.8 ± 20.3	< 0.0001
Height (cm)	147.1 ± 18.4	156.6 ± 11.6*	147.6 ± 18.1	147.2 ± 18.4	< 0.0001
Z-score Height	-0.14 ± 1.08	-0.10 ± 1.03	-0.15 ± 1.07	-0.16 ± 1.07	0.6179
BMI (kg/m^2)	22.4 ± 5.1	23.9 ± 4.8*	22.5 ± 5.1	22.4 ± 5.1	0.0001
Z-score BMI	1.3 ± 1.3	1.3 ± 1.3	1.3 ± 1.3	1.3 ± 1.3	1
Waist (cm)	74.6 ± 13.7	79.2 ± 11.8*	74.9 ± 13.5	74.6 ± 13.7	< 0.0001
Tanner stage					
1	144, 34%	30, 10%*	145, 33%	150, 33%	< 0.0001
2 & 3	143, 34%	129, 43%*	150, 34%	150, 33%	0.0141
4 & 5	138, 32%	138, 46%*	149, 34%	149, 33%	0.0001
BMI category					
Normal weight	143, 34%	89, 30%	146, 33%	150, 33%	0.2586
Overweight	134, 32%	103, 35%	148, 33%	149, 33%	0.4001
Obesity	148, 35%	105, 35%	150, 34%	150, 33%	0.2586
DXA measurements					
BMC (kg)	1.7 ± 0.7	2.0 ± 0.5	1.7 ± 0.7	1.7 ± 0.7	< 0.0001
Total FM (kg)	18.1 ± 9.6	21.0 ± 9.0	18.2 ± 9.5	18.0 ± 9.5	< 0.0001
FFM (kg)	32.4 ± 12.5	38.2 ± 10.3	32.7 ± 12.5	32.5 ± 12.6	< 0.0001
Lean mass (kg)	30.7 ± 11.8	36.1 ± 9.8	31.0 ± 11.8	30.8 ± 11.9	< 0.0001
ALM (kg)	13.8 ± 6.1	16.6 ± 5.1	14.0 ± 6.1	13.9 ± 6.1	< 0.0001
mBIA measurements					
FM (kg)	14.8 ± 8.9	17.4 ± 8.3	15.3 ± 9.1	15.1 ± 9.2	0.0001
FFM (kg)	35.7 ± 13.2	41.9 ± 10.9	35.7 ± 12.7	35.6 ± 12.6	< 0.0001
TBW (kg)	26.0 ± 8.9	29.9 ± 7.0	25.8 ± 8.8	25.5 ± 8.4	0.014
ASMM (kg)	14.1 ± 6.2	16.6 ± 5.0	14.0 ± 6.0	-	<0.0001
Trunk FM (kg)	8.8 ± 4.5	10.4 ± 5.1	8.9 ± 5.3	8.9 ± 5.5	0.0001
Phase angle	4.9 ± 0.6	5.0 ± 0.6	5.7 ± 0.7	5.7 ± 0.7$^+$	<0.0001
R50 (Ω)	749.4 ± 114.6	714.0 ± 97.6	697.9 ± 106.9	696.0 ± 109.7$^+$	<0.0001
Xc50 (Ω)	-63.4 ± 7.5	-62.0 ± 7.1	-68.6 ± 7.7	-68.9 ± 7.8$^+$	<0.0001

* Different group. $^+$Right side of the body. Abbreviations: BMI, body mass index; DXA, dual x-ray absorptiometry; BMC, bone mineral content; FM, fat mass; FFM, fat-free mass; ALM, appendicular lean mass; mBIA, multifrequency bioelectrical impedance analysis; TBW, total body water; ASMM, appendicular skeletal muscle mass; R, resistance, Xc: reactance.
